# Interaction of Natural Dietary and Herbal Anionic Compounds and Flavonoids with Human Organic Anion Transporters 1 (SLC22A6), 3 (SLC22A8), and 4 (SLC22A11)

**DOI:** 10.1155/2013/612527

**Published:** 2013-03-21

**Authors:** Li Wang, Douglas H. Sweet

**Affiliations:** Department of Pharmaceutics, Virginia Commonwealth University, 410 N 12th Street, Richmond, VA 23298, USA

## Abstract

Active components of complementary/alternative medicines and natural supplements are often anionic compounds and flavonoids. As such, organic anion transporters (OATs) may play a key role in their pharmacokinetic and pharmacological profiles, and represent sites for adverse drug-drug interactions. Therefore, we assessed the inhibitory effects of nine natural products, including flavonoids (catechin and epicatechin), chlorogenic acids (1,3- and 1,5-dicaffeoylquinic acid), phenolic acids (ginkgolic acids (13 : 0), (15 : 1), and (17 : 1)), and the organic acids ursolic acid and 18**β**-glycyrrhetinic acid, on the transport activity of the human OATs, hOAT1 (SLC22A6), hOAT3 (SLC22A8), and hOAT4 (SLC22A11). Four compounds, 1,3- and 1,5-dicaffeoylquinic acid, ginkgolic acid (17 : 1), and 18**β**-glycyrrhetinic acid, significantly inhibited hOAT1-mediated transport (50 **μ**M inhibitor versus 1 **μ**M substrate). Five compounds, 1,3- and 1,5-dicaffeoylquinic acid, ginkgolic acids (15 : 1) and (17 : 1), and epicatechin, significantly inhibited hOAT3 transport under similar conditions. Only catechin inhibited hOAT4. Dose-dependency studies were conducted for 1,3-dicaffeoylquinic acid and 18**β**-glycyrrhetinic acid on hOAT1, and IC_50_ values were estimated as 1.2 ± 0.4 **μ**M and 2.7 ± 0.2 **μ**M, respectively. These data suggest that 1,3-dicaffeoylquinic acid and 18**β**-glycyrrhetinic acid may cause significant hOAT1-mediated DDIs *in vivo*; potential should be considered for safety issues during use and in future drug development.

## 1. Introduction

In concert with our growing knowledge of drug transporter expression and function, transporter-mediated drug-drug interactions (DDIs) are being increasingly identified by numerous *in vitro* and *in vivo* studies [1, 2]. Recently, government agencies in the United States (Food and Drug Administration) and Europe (European Medicines Agency), as well as the pharmaceutical industry have acknowledged that transporters play a key role in the absorption, distribution, and excretion of many clinical therapeutics. Organic anion transporter 1 (OAT1; SLC22A6), OAT3 (SLC22A8), and OAT4 (SLC22A11) are among the transporters identified, thus far, to impact the pharmacokinetics, and hence dosing, efficacy, and toxicity, of some drugs. OAT1 and OAT3, expressed in the basolateral membrane of renal proximal tubule cells in both humans and preclinical species, function as key mediators for organic solute flux from blood to the glomerular filtrate [1, 2]. Additionally, OAT4, which is exclusively expressed in the apical membrane of human proximal tubules, reabsorbs anionic compounds from the urine [1]. Further, many endogenous substances, including hormones, neurotransmitters, and toxins, have been identified as substrates and/or inhibitors of OATs [1, 2]. Thus, the potential clinical significance of OAT-mediated DDIs is firmly recognized. 

Although many studies have exhibited OAT-mediated DDIs for synthesized drugs, relatively little is known about the potential interaction between OATs and natural products, including various organic anions, phenolic acids, and flavonoids found in herbal supplements and food. Several dietary flavonoids and their metabolic conjugates (e.g., sulfates and glucuronides) were identified as potent inhibitors and/or substrates of human (h) OAT1, hOAT3, and hOAT4 [3, 4]. Phenolic acids, for example, contained in the widely used Chinese herbal medicine Danshen (*Salvia miltiorrhiza*) or common berries such as strawberries or blueberries, were similarly demonstrated to interact with these three transporters [5, 6]. These studies highlight the strong potential for hOAT-mediated natural product-drug interaction and the need to investigate further anionic compounds and flavonoids that are found in popular complementary/alternative medicines and natural supplements.

Catechin and epicatechin are major components of tea products, possessing antioxidative and purported anticancer properties [7, 8]. 1,3- and 1,5-dicaffeoylquinic acids are two enantiomers of dicaffeoylquinic acid with various pharmacological effects, which are widely distributed in plants (e.g., coffee beans, sweet potato leaves, and fennels) [9]. Indeed, 1,3-dicaffeoylquinic acid (also known as cynarin) is being actively investigated for its anti-HIV and immunosuppressive properties [10]. Extracts of *Ginkgo biloba* have gained popularity as a herbal supplement because they are believed to improve mental sharpness and memory, while slowing brain aging, and were hoped to be effective in relieving symptoms associated with Alzheimer's disease. Regardless, it is well recognized that several ginkgolic acids, particularly (13 : 0), (15 : 1), and (17 : 1) (designating the number of carbon atoms in the alkyl side chain), can produce severe allergic, cytotoxic, mutagenic, carcinogenic, and genotoxic effects [11]. Licorice root (*Radix Glycyrrhizae*) is employed to relieve a number of maladies including stomach ulcers, colic, chronic gastritis, sore throat, bronchitis, osteoarthritis, liver disorders, malaria, and tuberculosis. One major component of *Radix Glycyrrhizae* preparations, glycyrrhizin, gives rise to two bioactive metabolites, 18*α*- and 18*β*-glycyrrhetinic acid, thought to play a role in these beneficial effects [12]. Ursolic acid is a pentacyclic triterpene acid occurring naturally in herbs and fruits. It exhibits anti-inflammatory and anticarcinogenic activities [13] and is marketed as a herbal supplement to promote weight loss. Since these compounds have been identified as major components of first-line/complementary/alternative medicines, foods, and beverages, humans can be exposed to these compounds through clinical therapies and the daily diet. Many of these compounds have shown systemic exposure in humans (Table 1). Based on their chemical structures and previous studies, these compounds have the potential to interact with OATs. Thus, OAT-mediated DDIs may occur *in vivo* when combined with known OAT substrates and such information should prove helpful in guiding the safe use and development of products that contain these compounds.

Therefore, the purpose of the present study was to investigate the inhibitory potential of these nine compounds, catechin, epicatechin, 1,3- and 1,5-dicaffeoylquinic acids, ginkgolic acids (13 : 0), (15 : 1) and (17 : 1), 18*β*-glycyrrhetinic acid, and ursolic acid, on hOAT1-, hOAT3- and hOAT4-mediated transport activities. In addition to generating transporter specific inhibition profiles, dose-response studies were conducted for potent inhibitors in order to derive inhibitory constants (IC_50_ and *K*_*i*_ values) to aid evaluation of their potential for clinical OAT-mediated DDIs.

## 2. Material and Methods 

### 2.1. Purified Chemicals

**Figure 1:**
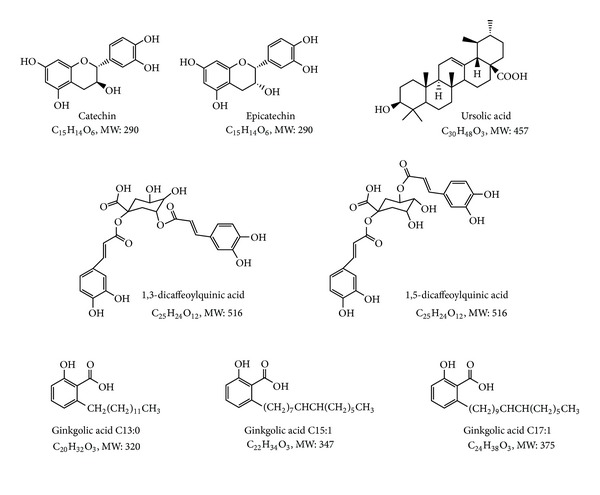
Chemical structures of compounds investigated in this study. MW: molecular weight.

Catechin, 1,3- and 1,5-dicaffeoylquinic acids, epicatechin, ginkgolic acids (13 : 0), (15 : 1) and (17 : 1), and ursolic acid (all ≥97% purity) were purchased from Tauto Biotech (Shanghai, China). 18*β*-glycyrrhetinic acid (≥97% purity) was obtained from Sigma-Aldrich (St. Louis, MO). The chemical structures of these compounds are shown in Figure 1. Tritiated *p*-aminohippuric acid ([^3^H]PAH) and estrone sulfate ([^3^H]ES) were purchased from PerkinElmer Life and Analytical Sciences (Waltham, MA), and unlabeled PAH, ES, and probenecid were purchased from Sigma-Aldrich (St. Louis, MO). 

### 2.2. Cell Culture

Derivation and culture of stably transfected Chinese hamster ovary (CHO) cells expressing hOAT1 (CHO-hOAT1) and hOAT4 (CHO-hOAT4), stably transfected human embryonic kidney 293 (HEK) cells expressing hOAT3 (HEK-hOAT3), and their corresponding empty vector transfected background control cell lines, have been described previously [6, 14–16].

### 2.3. Cell Accumulation Assays

The cell accumulation assay protocol was performed as described in our recent publications with minor modifications [14, 17]. In brief, 2 × 10^5^ cells/well were seeded in 24-well tissue culture plates and grown in the absence of antibiotics for two days. For transport experiments cells were preincubated in transport buffer without substrates or inhibitors for 10 min after which time the buffer was replaced with 400 *μ*L of fresh transport buffer containing 1 *μ*M [^3^H]PAH (0.5 *μ*Ci/mL) or [^3^H]ES (0.25 *μ*Ci/mL) with or without test compounds. After incubation for times specified in the figure legends, cellular uptake was quenched by quickly rinsing each well three times with fresh ice-cold transport buffer. Cells were lysed with 1 M NaOH and radioactivity was measured with a liquid scintillation counter and reported as picomoles of substrate per milligram total protein. All intracellular uptake data were corrected for background accumulation in corresponding empty vector transfected control cells. Kinetic calculations were performed using GraphPad Prism Software version 5.0 (GraphPad Software Inc., San Diego, CA). The half maximal inhibitory concentrations (IC_50_) and inhibitory constants (*K*_*i*_) were calculated using nonlinear regression with the appropriate models. Results were confirmed by repeating all experiments at least three times with triplicate wells for each data point. For kinetic analysis, hOAT1 expressing cells showed linear PAH uptake for the initial 3 min with *K*_*m*_ = 15.4 *μ*M [15], while hOAT3 expressing cells exhibited linear ES uptake for the initial 1 min with *K*_*m*_ = 14.5 *μ*M [6].

### 2.4. Statistics

Values are expressed as mean ± S.D. or mean ± S.E.M. as indicated. Statistical differences were assessed using one-way analysis of variance (ANOVA) followed by *post hoc* analysis with Dunnett's *t*-test (*α* = 0.05).

## 3. Results

### 3.1. Inhibition of hOAT1 by Natural Anionic Compounds and Flavonoids

**Figure 2:**
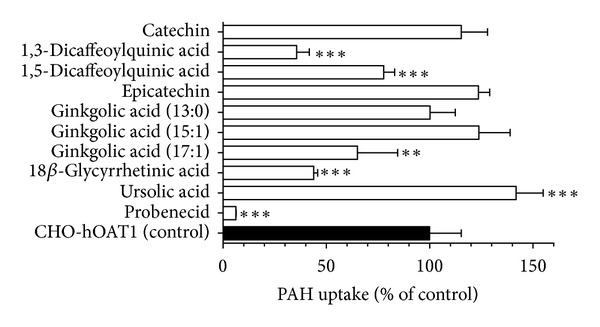
Inhibitory effects of the nine test compounds on hOAT1-mediated PAH uptake. Ten-minute uptake of [^3^H]PAH (1 *μ*M) was measured in CHO-hOAT1 cells in the absence and presence of test compounds (50 *μ*M) or probenecid (1000 *μ*M). Data represent hOAT1-mediated transport specifically, that is, data have been corrected for background PAH accumulation measured in empty vector transfected cells. Values are mean ± S.D. of triplicate values from a representative experiment. ** denotes  *P* < 0.01, and *** denotes  *P* < 0.001 as determined by one-way ANOVA followed by Dunnett's *t*-test.

**Figure 3:**
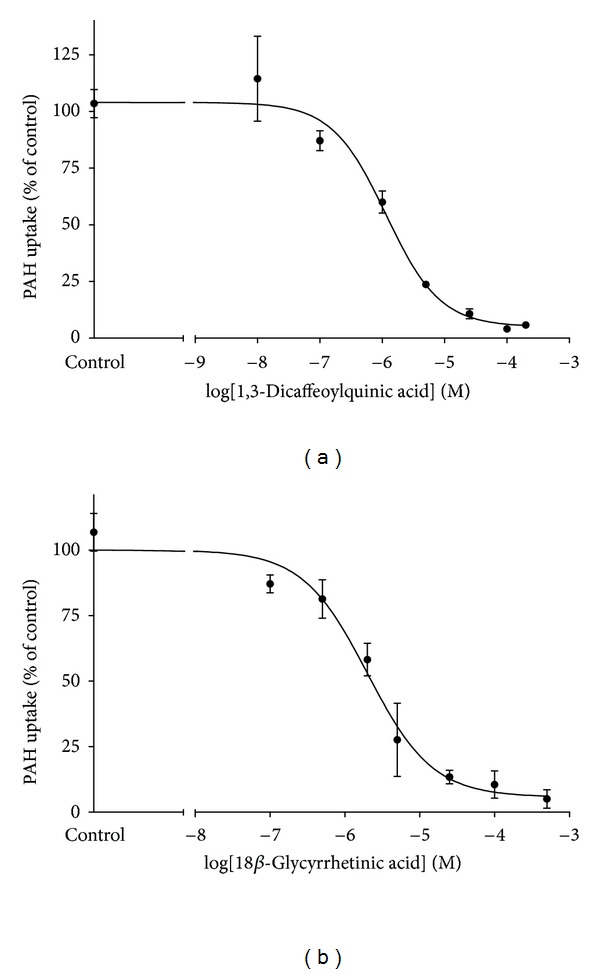
Dose-response effects for 1,3-dicaffeoylquinic acid and 18*β*-glycyrrhetinic acid on hOAT1-mediated PAH transport. One-minute uptake of [^3^H]PAH (1 *μ*M) in CHO-hOAT1 cells was measured in the presence of increasing concentrations (10^−7^ to 5 × 10^−4^  M) of test compounds. Data were corrected by subtracting background PAH accumulation measured in empty vector transfected cells. IC_50_ values were determined with nonlinear regression and the “log(inhibitor) versus response” model using GraphPad Prism software. Experiments were repeated three times with triplicate samples and graphs shown are from representative experiments with values plotted as mean ± S.D. (*n* = 3). The data were used to generate mean IC_50_ ± S.E.M. estimates.

Approximately threefold greater accumulation of PAH was observed in CHO-hOAT1 cells (6.30 ± 0.97 pmol mg protein^−1^ 10 min⁡^−1^) than in empty vector transfected background control cells (2.12 ± 0.19 pmol mg protein^−1^ 10 min⁡^−1^). The hOAT1-mediated PAH accumulation was almost completely inhibited by probenecid (Figure 2). The nine test compounds, catechin, 1,3- and 1,5-dicaffeoylquinic acid, epicatechin, ginkgolic acids (13 : 0), (15 : 1) and (17 : 1), 18*β*-glycyrrhetinic acid, and ursolic acid, were assessed for inhibitory effects on hOAT1-mediated uptake (Figure 2). 1,3- and 1,5-Dicaffeoylquinic acid (64% and 22% inhibition, resp.), ginkgolic acid (17 : 1) (42% inhibition), and 18*β*-glycyrrhetinic acid (56% inhibition) each significantly inhibited hOAT1-mediated PAH uptake at 50-fold excess. Ursolic acid produced a significant stimulation of uptake and the other compounds were without effect. Since 1,3-dicaffeoylquinic acid and 18*β*-glycyrrhetinic acid produced inhibition greater than 50%, they were further characterized by dose-response studies (0.01–500 *μ*M, shown in Figure 3). Estimated IC_50_ values were 1.2 ± 0.4 *μ*M for 1,3-dicaffeoylquinic acid and 2.7 ± 0.2 *μ*M for 18*β*-glycyrrhetinic acid. Numerous studies investigating the mode of inhibition produced on hOAT1 and hOAT3 for a broad array of compounds have demonstrated the interaction to be competitive [5, 18–22]. Therefore, assuming competitive inhibition of hOAT1, inhibition constants (*K*_*i*_) were estimated as 1.1 ± 0.2 *μ*M for 1,3-dicaffeoylquinic acid and 2.5 ± 0.2 *μ*M for 18*β*-glycyrrhetinic acid.

### 3.2. Inhibition of hOAT3 by Natural Anionic Compounds and Flavonoids

**Figure 4:**
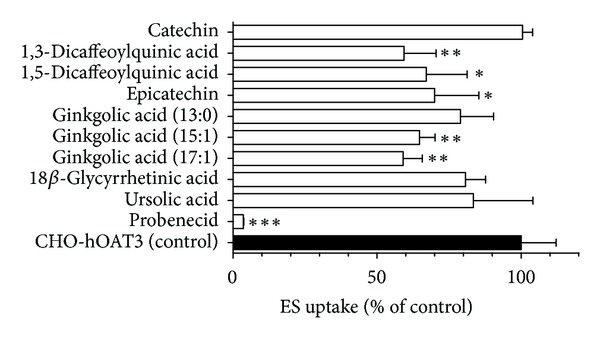
Inhibitory effects of the nine test compounds on hOAT3-mediated ES uptake. Ten-minute uptake of [^3^H]ES (1 *μ*M) was measured in HEK-hOAT3 cells in the absence and presence of test compounds (50 *μ*M) or probenecid (1000 *μ*M). Data represent hOAT3-mediated transport specifically, that is, data have been corrected for background ES accumulation measured in empty vector transfected cells. Values are mean ± S.D. of triplicate values from a representative experiment. * denotes  *P* < 0.05, ** denotes  *P* < 0.01, and ***  denotes  *p* < 0.001 as determined by one-way ANOVA followed by Dunnett's *t*-test.

Human OAT3 expressing cells showed about 4-fold greater accumulation of ES as compared to background control cells (10.6 ± 0.5 versus 2.6 ± 0.3 pmol mg protein^−1^ 10 min⁡^−1^, resp.). Similar to hOAT1, hOAT3-mediated ES uptake was completely (>96% inhibition) blocked by probenecid (Figure 4). Five of the compounds, 1,3- and 1,5-dicaffeoylquinic acid, epicatechin, and ginkgolic acids (15 : 1) and (17 : 1), significantly inhibited hOAT3-mediated transport at 50-fold excess (Figure 4). 1,3-Dicaffeoylquinic acid and ginkgolic acid (17 : 1) exhibited 41% inhibition, while 30–35% reduction of hOAT3-mediated ES uptake was observed for 1,5-dicaffeoylquinic acid, epicatechin, and ginkgolic acid (15 : 1). Catechin, 18*β*-glycyrrhetinic acid, and ursolic acid failed to produce significant inhibition. Based on the level of inhibition observed, IC_50_ values for all of these compounds would be greater than 50 *μ*M, much higher than clinically relevant concentrations (Table 1). Therefore, further dose-response studies were not performed.

### 3.3. Inhibition of hOAT4 by Natural Anionic Compounds and Flavonoids

**Figure 5:**
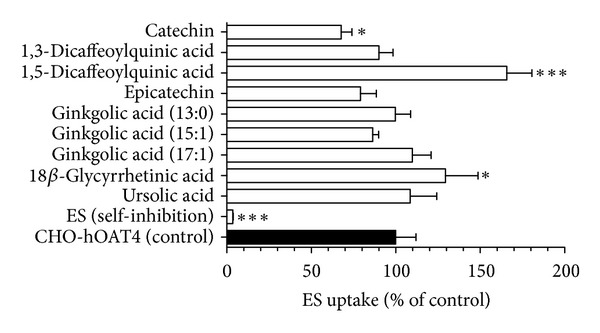
Inhibitory effects of the nine test compounds on hOAT4-mediated ES uptake. Ten minute uptake of [^3^H]ES (1 *μ*M) was measured in CHO-hOAT4 cells in the absence and presence of test compounds (50 *μ*M) or ES (1000 *μ*M for self-inhibition). Data represent hOAT4-mediated transport specifically, that is, data have been corrected for background ES accumulation measured in empty vector transfected cells. Values are mean ± S.D. of triplicate values from a representative experiment. * denotes  *P* < 0.05, and *** denotes  *P* < 0.001 as determined by one-way ANOVA followed by Dunnett's *t*-test.

Stably transfected hOAT4-expressing cells showed ~18 fold higher accumulation of ES than background control cells (42.5 ± 5.1 versus 2.4 ± 0.2 pmol mg protein^−1^ 10 min⁡^−1^, resp.). Human OAT4-mediated ES uptake was virtually completely blocked (>96% inhibition) by self-inhibition (Figure 5). Catechin (50 *μ*M) was the only compound that showed a significant inhibitory effect (32%) on hOAT4 (Figure 5). 1,5-Dicaffeoylquinic acid and 18*β*-glycyrrhetinic acid produced significant stimulation of ES uptake. Accordingly, the IC_50_ value for catechin on hOAT4 transport activity should be greater than 50 *μ*M and no further kinetic studies were performed.

## 4. Discussion

Literally hundreds of therapeutic and endogenous compounds have been demonstrated to interact with OATs [1, 2]. Precisely because of their broad, polyspecific nature, OATs are likely sites for DDIs *in vivo*. As a consequence, they have been identified worldwide as a family of “clinically relevant” transporters in recent government guidances to the pharmaceutical industry [1, 2]. Unlike Western drugs, the pharmacokinetic and pharmacological properties of botanical drug products and natural supplements have not been well investigated. Being naturally derived, people often view these compounds as “safe” and without potential for causing adverse events. However, there is increasing evidence demonstrating potent interactions between components of natural products and OATs, resulting in possible toxicity and DDIs [1, 2]. For example, aristolochic acid, a potent nephrotoxic contaminant sometimes found in certain Chinese herbal therapies, was identified as a high affinity substrate of murine Oat1, Oat2, and Oat3, as well as a potent inhibitor of hOAT1, hOAT3, and hOAT4 [26, 27]. Additional studies have demonstrated the OAT-mediated DDI potential for common flavonoid and phenolic acid components of foods and herbal medicines [3–6]. These investigations showed that many dietary/phytomedicine-derived organic acids and flavonoids have a high inhibitory potency for hOAT1, hOAT3, and/or hOAT4 and, according to proposed guidelines established by the FDA and EMA, high likelihood to result in significant DDIs *in vivo* [1–6]. Therefore, further studies on OAT-mediated natural product-drug interactions are needed in order to establish informed safety and efficacy profiles for botanical products.

In the present study, we characterized the interactions of nine chemically diverse compounds including flavonoids, chlorogenic acids, phenolic acids, and other organic acids, found as major dietary or phytomedicine components, with three human OATs: hOAT1, hOAT3, and hOAT4. As illustrated, most of the compounds produced significant inhibition of hOAT1 and hOAT3 at a 50-fold excess concentration (Figures 1 and 3). In marked contrast, only catechin significantly inhibited hOAT4 under this condition (Figure 5). Interestingly, ursolic acid caused a significant stimulation of hOAT1-mediated PAH uptake while it was without effect on either hOAT3 or hOAT4 transport activity. Similarly, 1,5-dicaffeoylquinic acid and 18*β*-glycyrrhetinic acid appeared to significantly stimulate hOAT4 transport while decreasing hOAT1- and hOAT3-mediated uptake activities. Such sporadic transporter stimulation/inhibition has been previously reported in the literature for other compounds. For example, for fluoroquinolone antimicrobials, ciprofloxacin inhibited hOAT3, but stimulated hOAT1 [14], and sparfloxacin was reported to stimulate multidrug resistance-associated protein 2 activity [28]. Such stimulation of transport activity *in vitro* also has been reported for steroids, chemotherapeutic agents, and nonsteroidal anti-inflammatory drugs [14, 28–30]. One potential explanation is allosteric binding of the compounds to the transporters with subsequent alteration of substrate affinity [28, 29]. However, these transporters share a high degree of sequence identity, and no readily discernible structural differences corresponding to such a binding site are apparent, and these effects do not exhibit a consistent pattern among the hOATs. Whether these effects will be observed *in vivo* is yet to be determined; however, in such an instance increased (versus decreased) elimination and shortened (versus lengthened) terminal half-life of victim drugs could occur causing loss of efficacy.

The FDA guidance on drug interaction studies proposed using the DDI index, calculated as the ratio of unbound *C*_max⁡_ over IC_50_ or *K*_*i*_, as an indicator of the assessment of a compound's DDI potential, with a value greater than 0.1 indicating the potential need to perform an *in vivo* DDI study for an investigational drug. In the current study, 18*β*-glycyrrhetinic acid exhibited strong inhibition of hOAT1 with an estimated IC_50_ of 2.7 ± 0.2 *μ*M or *K*_*i*_ of 2.5 ± 0.2 *μ*M (Figures 1 and 2). Human exposure studies reported *C*_max⁡_ values ranging from 0.11 to 2.9 *μ*M (Table 1), perhaps due to interindividual variability, different doses, and/or dosing regimen (single dose versus repeated dose). As such, the maximum DDI for 18*β*-glycyrrhetinic acid is ~1.1, although this value does not account for plasma protein binding as this is unknown. However, if we assume it to be highly plasma protein bound, for example, 90%, the DDI index would be 0.12, meeting the FDA guidance threshold of 0.1. Therefore, 18*β*-glycyrrhetinic acid may affect hOAT1-mediated renal elimination of coadministered therapeutics that are hOAT1 substrates.

1,3-Dicaffeoylquinic acid is a major component found in artichoke and *Echinacea purpurea *[31, 32]. Pharmacological studies demonstrated that it exhibits antimicrobial and antioxidant activity [31, 33]. Moreover, it has garnered increased interest because of its anti-HIV and immunosuppressive properties. 1,3-Dicaffeoylquinic acid blocked HIV-1 integrase activity, leading to interference of insertion of viral DNA into the genome of the victim cell [10]. Another study demonstrated that it inhibited the interaction between CD28 of T-cell receptor and CD80 of antigen presenting cells, blocking “signal 2” of T-cell activation [32]. Due to its potential development as a therapeutic agent, systemic exposure in clinical applications may reach high levels. In the present study, 1,3-dicaffeoylquinic acid showed high affinity with hOAT1 (IC_50_ = 1.2 ± 0.4 *μ*M; *K*_*i*_ = 1.1 ± 0.2 *μ*M). Therefore, the dose-systemic exposure relationship and plasma protein binding should be elucidated as part of the future drug development process for this compound.

It has been reported that flavonoids can interact with OATs [3]. Therefore, catechin and epicatechin, two flavonoid components from green tea, were investigated for potential interaction with hOAT1, hOAT3, and hOAT4. Both compounds exhibited significant inhibition of hOAT4 and hOAT3 when present at a 50-fold excess; however no inhibition was observed for hOAT1. Because of low reported clinical plasma concentrations (Table 1), these flavonoids were determined to be unlikely to cause DDIs after normal consumption of tea products. However, OATs might promote entry of these antioxidants into renal proximal tubules, providing a nephroprotective effect.

In summary, we investigated the potential interaction of nine flavonoids, phenolic acids, chlorogenic acids, and other organic acids with hOAT1, hOAT3, and hOAT4. Among the examined compounds, 1,3-dicaffeoylquinic acid and 18*β*-glycyrrhetinic acid showed marked affinity with hOAT1. In humans, systemic exposure of 18*β*-glycyrrhetinic acid may induce significant hOAT1-associated DDIs. Future *in vivo* interaction studies between 18*β*-glycyrrhetinic acid or 1,3-dicaffeoylquinic acid and clinical therapeutics known to be hOAT1 substrates may be necessary to establish safety guidelines for use of pharmaceutical products containing these compounds to avoid potential DDIs. 

## Figures and Tables

**Table 1:** Maximum plasma concentration (*C*_max⁡_) reported in human subjects.

Compound	*C*_max⁡_ (*μ*M)	Route of administration	Dose (mg)	Reference
Epicatechin	0.20	Oral	37	[7]
1,5-Dicaffeoylquinic acid	0.14	Oral	600	[23]
18*β*-Glycyrrhetinic acid	0.11–2.9^a^	Oral	130–144 (glycyrrhizin)	[12, 24, 25]
Ursolic acid	7.4	i.v.	186	[13]

^a^Assuming plasma concentration of 18*α*- and 18*β*-glycyrrhetinic acid is about 1 : 3 as reported in [12].
